# Uncovering Biologically Coherent Peripheral Signatures of Health and Risk for Alzheimer’s Disease in the Aging Brain

**DOI:** 10.3389/fnagi.2018.00390

**Published:** 2018-11-29

**Authors:** Brandalyn C. Riedel, Madelaine Daianu, Greg Ver Steeg, Adam Mezher, Lauren E. Salminen, Aram Galstyan, Paul M. Thompson, Alzheimer’s Disease Neuroimaging Initiative

**Affiliations:** ^1^Imaging Genetics Center, Mark and Mary Stevens Neuroimaging and Informatics Institute, University of Southern California, Marina del Rey, CA, United States; ^2^USC Information Sciences Institute, Marina del Rey, CA, United States; ^3^Departments of Neurology, Psychiatry, Radiology, Engineering, and Pediatrics, University of Southern California, Los Angeles, CA, United States

**Keywords:** information theory, machine learning, Alzheimer’s disease, plasma biomarkers, neuroimaging

## Abstract

Brain aging is a multifaceted process that remains poorly understood. Despite significant advances in technology, progress toward identifying reliable risk factors for suboptimal brain health requires realistically complex analytic methods to explain relationships between genetics, biology, and environment. Here we show the utility of a novel unsupervised machine learning technique – Correlation Explanation (CorEx) – to discover how individual measures from structural brain imaging, genetics, plasma, and CSF markers can jointly provide information on risk for Alzheimer’s disease (AD). We examined 829 participants (*M*_age_: 75.3 ± 6.9 years; 350 women and 479 men) from the Alzheimer’s Disease Neuroimaging Initiative database to identify multivariate predictors of cognitive decline and brain atrophy over a 1-year period. Our sample included 231 cognitively normal individuals, 397 with mild cognitive impairment (MCI), and 201 with AD as their baseline diagnosis. Analyses revealed latent factors based on data-driven combinations of plasma markers and brain metrics, that were aligned with established biological pathways in AD. These factors were able to improve disease prediction along the trajectory from normal cognition and MCI to AD, with an area under the receiver operating curve of up to 99%, and prediction accuracy of up to 89.9% on independent “held out” testing data. Further, the most important latent factors that predicted AD consisted of a novel set of variables that are essential for cardiovascular, immune, and bioenergetic functions. Collectively, these results demonstrate the strength of unsupervised network measures in the detection and prediction of AD.

## Introduction

Alzheimer’s disease (AD) affects approximately 10% of the American population over age 65, and several lines of evidence suggest that there is an extended preclinical phase during which treatments are most likely to be effective (Brookmeyer et al., 2011; Alzheimer’s Association, 2018). The growing list of potential biomarkers (from neuroimaging, genetics, proteomics, and cognition) offers increasing potential for early diagnosis of AD and better prognosis of age-associated diseases. The most widely accepted etiological model for AD suggests there is a temporal order in brain changes that are characteristic of AD pathology, and map onto the phenotypic profile of amnestic cognitive decline (Braak and Braak, 1991; Jack et al., 2016).

Accumulation of beta-amyloid “plaques” in the brain has been traditionally identified as a hallmark of AD that typically begins decades before the onset of clinical symptoms. Amyloid accumulation can be measured using positron emission tomography (PET) with amyloid tracers, or through cerebrospinal fluid (CSF) levels of Aβ1-42 (Hardy and Allsop, 1991; McKhann et al., 2011; Jack et al., 2016). The relationship between amyloid aggregation and clinical symptoms is believed to result from amyloid-induced neuronal injury and subsequent degeneration through disruption of the tau protein – the key component of neurofibrillary tangles (Mudher and Lovestone, 2002; Hampel et al., 2010; McKhann et al., 2011). Neuronal and synaptic loss trigger an atrophic state in the brain that is mirrored by decline in glucose metabolism in the temporal and parietal cortices, and increased accumulation of CSF tau and phosphorylated-tau (Brookmeyer et al., 2007; Mosconi et al., 2008; Mapstone et al., 2014). These neurologic changes have a detrimental impact on cognition and daily living and are often exacerbated among individuals at genetic risk for AD (e.g., positive for at least one APOE-ε4 allele) (Corder et al., 1993). Unfortunately, decades of research focusing on tau and amyloid as prodromal biomarkers for AD have not yet yielded measures that correlate well with patient health, suggesting that these classical disease hallmarks may not be ideal biomarkers of AD.

Machine learning offers a promising alternative to traditional research methods for improving prediction of disease and identifying clinically relevant signatures of risk (Jensen and Bateman, 2011). For example, Ray et al. (2007) used a set of 18 plasma signaling proteins to discriminate cognitively normal controls from people with AD and other forms of dementia with 89% classification accuracy. Similarly, Doecke et al. (2012) classified AD patients from healthy controls with 85% accuracy using a combination of plasma inflammatory markers, participant demographics, and clinical information (Doecke et al., 2012). Although a thorough review is beyond the scope of this paper, most work using machine learning algorithms in AD research has focused on cross-sectional measures, despite increasing motivation to investigate biomarkers that predict conversion to AD in cognitively normal individuals or those with mild cognitive impairment (MCI). Pertinent to the present study, Westman et al. (2012) reported classification accuracy of only 58.6–66.4% for distinguishing people with stable MCI from those who progressed to AD over a 12–36-month period using a combination of structural MRI and CSF measures. That study used supervised methods, did not explore contributions of plasma to classification, and sought to classify MCI groups using an AD vs. control model.

To improve these results and better understand AD pathogenesis, it is important to determine the best set of variables (features) for predicting clinical progression, and how these features interact during specific stages of disease progression. Machine learning methods can identify predictors of diagnosis and prognosis, but most methods (‘supervised’ algorithms) require diagnostic categories to be defined *a priori* and this is contrary to what is known about the AD continuum. Systems biology approaches aim to address this by moving beyond discrete biomarkers of disease to biological networks that may better reflect disease complexity (Padmanabhan et al., 2017). Here, we introduce a novel method – Correlation Explanation (CorEx) – to overcome several limitations of traditional machine learning techniques. CorEx uses information theory and unsupervised learning to identify combinations of biomarkers across a range of diverse data types that maximize predictive power for disease progression in AD. Using this approach, we aim to better understand predictors of decline in AD in a tractable and principled way. Here we used CorEx to study the discriminative value of over 400 genetic, plasma proteomic, CSF, imaging, and demographic measures from 829 participants from phase 1 of the Alzheimer’s Disease Neuroimaging Initiative (ADNI-1). We hypothesized that CorEx would discover latent factors across various data types to enhance predictive accuracy for individuals at high risk for disease progression. We used longitudinal information on the diagnosis of each individual to test models that distinguished (1) stable cognitively normal (CN) individuals from CN and MCI individuals who progress to AD, (2) stable MCI, from CN and MCI individuals who progress to AD, (3) any diagnosis of AD from individuals who do not have or progress to AD over the time frame of the study. For each of these prediction problems, we determined the feature relevance. We hypothesized that using CorEx to boost predictive power, a latent factor representing the classical hallmarks of AD - namely APOE4 and CSF Aβ1-42 and tau levels - would be among the most consistent factors for predicting clinical progression across analyses. We further hypothesized that predictive latent factors would correspond to brain atrophy and cognitive decline over time.

## Materials and Methods

### Participants

Data were collected from 829 individuals (350 women, 479 men) participating in ADNI-1 – a longitudinal study of biomarkers of AD. Diagnosis of probable AD was based on the NINCDS-ADRDA Alzheimer’s Criteria (McKhann et al., 1984). Inclusion and exclusion criteria may be found in Petersen et al. (2010). All individuals were free from significant neurologic disease other than Alzheimer’s disease. Visits occurred once every 6 months for the first 2 years of the study, and once annually thereafter. All individuals included here had plasma collected, completed a cognitive battery of tests, and underwent a brain scan for their baseline and follow-up visits, where applicable. All ADNI-1 data are publicly available online^1^ (Weiner et al., 2017). Study procedures were conducted according to the Good Clinical Practice guidelines, the Declaration of Helsinki, and the US 21 CFR Part 50-Protection of Human Subjects, and Part 56-Institutional Review Boards (IRB). Written informed consent was obtained from all participants. All study procedures were approved by the local and participating IRBs of the ADNI study.

### Biomarker Quantification and Analysis

An extensive panel of 203 laboratory tests was collected for all participants at the baseline visit. The panel consisted of plasma protein markers from the Luminex XMAP platform by Rules-Based Medicine (Myriad RBM, Austin, TX, United States). Proteins included markers of liver function, cytokines, lipoproteins, oxidative stress, growth factors, hormone levels, glucose metabolism, and amyloid and tau levels, among others. Full protocol details are available through the ADNI website^2^. We examined CSF levels from lumbar punctures of tau, phosphorylated tau 1–81, and amyloid-β-1-42 levels collected at the baseline visit. Quality control procedures have been described by the ADNI Biomarker Core (Shaw et al., 2009; Soares et al., 2012). Genomic analyses were completed according to the ADNI protocol. Our genetic marker of interest was APOE ε4 carrier status, coded as the number of ε4 alleles. For clarity below, we categorize measures into: (1) Demographics (height, weight, sex, age), (2) Hallmarks of AD pathology (CSF measures and APOE ε4 count), and (3) Plasma proteomics (all other measures, including a urine test of kidney functions).

### Scan Acquisition and Image Processing

All participants underwent whole-brain magnetic resonance imaging (MRI) on 1.5 T GE, Siemens, or Philips scanners at one of 59 sites across North America. A standardized MRI protocol was used across scanner platforms to ensure cross-site compatibility (Jack et al., 2008). A typical 1.5 T MR protocol involved a 3D sagittal MP-RAGE scan with repetition time (TR): 2400 ms, minimum full TE, inversion time (TI): 1000 ms, flip angle: 8°, 24 cm field of view, and a 192 × 192 × 166 acquisition matrix in the x-, y-, and z- dimensions, yielding a voxel size of 1.25 mm × 1.25 mm × 1.2 mm that was later reconstructed to 1-mm isotropic voxels. We used standard image preprocessing to correct for motion, intensity normalization, affine registration of volumes to MNI space, skull stripping, non-linear registration using the Gaussian Classifier Atlas (GCA), and brain parcellation.

Regions of interest (ROIs) included 68 cortical and 8 subcortical structures extracted from baseline images using FreeSurfer version 5.3 and the Desikan-Killiany atlas^3^ (Fischl et al., 2002). Cortical metrics included thickness and surface area, whereas subcortical structures were measured as regional volumes. All brain measures were analyzed separately between hemispheres. To address potential confounds, we regressed out the effects of age, sex, and education on surface area, thickness, and volume measures, as well as intra-cranial volume (ICV) on surface area and volume measures. All subsequent analyses use these residualized measures.

### Tensor-Based Morphometry Measures of Atrophy

MRI-derived measures of structural brain atrophy were computed by comparing each subject’s 1-year follow-up MRI scan to their baseline scan and measuring temporal lobe tissue loss and ventricular expansion using tensor-based morphometry (TBM). Registration of preprocessed follow-up scans to baseline scans was completed with a non-linear inverse-consistent elastic intensity-based registration algorithm, optimizing a joint cost-function from the mutual information and elastic deformation of the images (Hua et al., 2013). Then, representations of the degree of local contraction or expansion of the 3D registration from the 1-year scan to baseline, also known as the Jacobian determinant map, was computed at the voxel level. Values in these maps represent relative tissue volume differences expressed as positive or negative percentages of their baseline. We used the Jacobian determinant to assess morphometry in a bilateral temporal lobe region of interest as in Hua et al. (2013). As above, we extracted ventricular surfaces for each subject and registered the baseline and follow-up timepoints to determine ventricular expansion or contraction between scans (Hua et al., 2011; Gutman et al., 2013).

### Correlation Explanation

Correlation Explanation is an information-theoretic optimization method that constructs a low-dimensional hierarchy of latent factors that progressively explain non-linear dependencies in the observations *X*_1_…*X*_N_ as measured by maximizing the multivariate mutual information - also called *total correlation* (TC)^4^ (Ver Steeg and Galstyan, 2014, 2015).

$$TC(X_{1},...,X_{N})\equiv \overset{n}{\underset{i=1}{\Sigma }}H(X_{i})-H\left(X_{1},...,X_{N}\right)$$

The special case of two variables is more commonly known as mutual information (I) and is defined as the difference between the sum of the individual entropies (H) and the entropy of the variables considered together.

$$I(X_{1};X_{1})\equiv H(X_{1})+H(X_{1})-H\left(X_{1},X_{1}\right)$$

The dependence in the data as measured by TC(X) can be reduced or “explained” by conditioning on constructed factors *Y*_1_…*Y*_M_. The conditional TC goes to zero if all variables are independent after conditioning on *Y*. “Explanation” refers to this latter phenomenon, with the constructed factors containing all the information about causes of dependence in the data. In contrast to other cluster-based learning approaches, CorEx naturally decomposes information in a hierarchical way: lower layers capture more local relationships, and higher layers reflect more global interactions a framework in line with the ideas of network biology. An example construction of the CorEx framework is presented in Supplementary Figure 1. Additional details of the optimization parameters and CorEx framework are in Ver Steeg and Galstyan (2014, 2015) and Pepke and Ver Steeg (2017).

We used CorEx to discover shared information among plasma and demographic measures, and the hallmarks of AD (APOE4 and CSF Aβ1-42 and tau levels) across all participants. We separately applied CorEx to discover shared information within residualized brain measures. A total of 25 latent factors were chosen for each CorEx model. This number of factors represents the simplest explanation of data with the maximum multivariate mutual information across factors and optimal total correlation for the corresponding measures. For clarity, we refer to these as “CorEx plasma” and “CorEx brain.” Latent factor constructions resulted in generally non-overlapping groups of variables that were maximally informative and robust to noise. Values obtained for each factor represent a decomposition of common information and correspond to the maximum likelihood labels for that specific latent factor and particular participant (Ver Steeg, 2017).

### Cognitive Assessment

Cognition was measured using three commonly used neuropsychological tests that are sensitive to AD – the Mini-Mental State Examination (MMSE; Folstein et al., 1975), the Alzheimer’s Disease Assessment Scale-cognitive subscale 13 item (ADAS-Cog; Mohs, 1996), and the Clinical Dementia Rating scale Sum of Boxes (CDR-SOB) (Hughes et al., 1982). Total scores from each test were aggregated into a composite *z*-score for each participant at baseline and 1-year follow-up visits. Total scores on the CDR-SOB and ADAS-Cog were multipled by -1 prior to z transformation so that lower scores represented poorer performance across all cognitive tests. We chose this approach over individual domain scores to capture an index of cognitive function that would be less prone to bias related to measurement errors (Ayutyanont et al., 2014; Langbaum et al., 2015). Similar approaches have been used in prior work on neurodegenerative diseases (Cutter et al., 1999; Crane et al., 2012; Donohue et al., 2014). A longitudinal composite score was created by subtracting the baseline composite from the 1-year follow-up composite score. This longitudinal composite score was the primary outcome measure for all cognitive analyses.

### Diagnostic Groupings

We identified two stable groups of cognitively normal (CN-s) and MCI (MCI-s) participants that had at least 1-year follow-up and a stable diagnosis across all study visits. In ADNI-1, individuals were followed for repeat assessments for an average of 1–5 years. We also identified two progressive groups of CN (CN-p) and MCI (MCI-p) individuals at baseline who completed at least one subsequent visit and progressed to AD during follow-up. Individuals in the MCI group who reverted to CN status were excluded from analyses comparing stable and progressing individuals. Overall, we identified 22 CN-p and 211 MCI-p patients, compared to 165 CN-s and 147 MCI-s patients after a mean follow-up of 52.5 months. This difference represents a 42.8% prevalence rate of AD, corresponding to an annual conversion rate of 9.8%. Finally, we conducted a broad case-control analysis that compared those who received a diagnosis of AD at baseline or follow-up, to those who did not receive an AD diagnosis at any time point. Average follow-up was 49.5 months for CN and MCI groups.

### Approach

**FIGURE 1:**
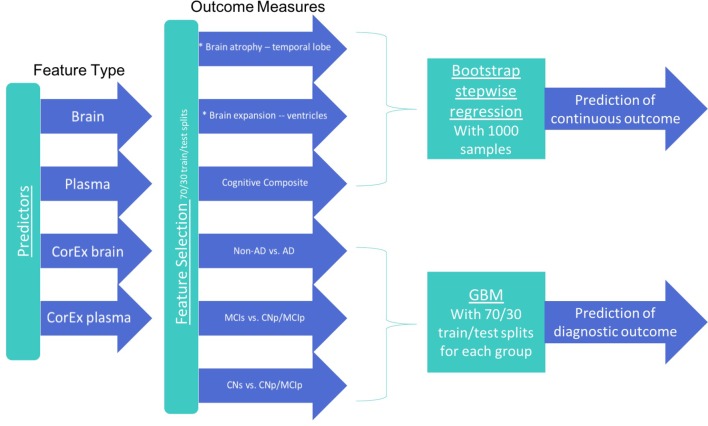
Features and analytic methods included in the current analyses. After constructing latent factors using CorEx within plasma and brain measures separately, we performed feature selection for each feature type to determine the top 10 features for each outcome measure and each feature type. We combined these top features across feature types within each corresponding outcome and either (1) performed disease prediction with gradient boosting classification or (2) used bootstrap stepwise regression to determine the importance of the top features for predicting the continuous outcome measures (i.e., cognition and TBM atrophy measures).

To better reflect the known temporal evolution of AD biomarkers in relation to each other and their relation to AD progression, our statistical analyses were designed to address three different classification tasks: (1) CN-s vs. CN-p/MCI-p, (2) MCI-s vs. CN-p/MCI-p, (3) non-AD vs. AD. For each group, we performed feature selection with a subset of participants using CorEx factors and the original features to determine the top 10 features in each class of variables (CorEx plasma, CorEx brain, plasma, brain measures). We then used those features with gradient boosting for diagnostic prediction, or bootstrap stepwise regression for cognitive and TBM outcomes. We outline the analytic steps in Figure 1. To determine the significance of model improvement using differing combinations of data types, we used the McNemar test to compare the top two models for predictions within each diagnostic subgroup.

### Feature Importance and Selection

We employed feature selection prior to our disease predictions to reduce training time and data dimensionality (Bell and Wang, 2000; Guyon and Elisseeff, 2003). For each subgroup analysis (CN-s vs. progressors (CN-p and MCI-p); MCI-s vs. progressors (CN-p and MCI-p); non-AD vs. AD), we applied a random split of the data according to diagnosis, using 70% for training, and the remaining 30% for hold-out testing. We then used an ensemble of machine learning methods to determine feature importance within each subgroup analysis (Seijo-Pardo et al., 2017). The ensemble approach used the following methods for binary predictions: General Linear Model (GLM), Gradient Boosting Machines (GBM), Treebag, Linear Discriminant Analysis (LDA), and K-Nearest Neighbors (KNN), while ensemble regression tasks included GLM, GBM, KNN, ridge regression, and the least absolute shrinkage and selection operator (LASSO) method. For each model, a weighted-average mean squared error was used across methods to estimate prediction error on unseen test data, and its reported association using results across 10-times repeated 10-fold cross-validation (Pacławski et al., 2015). For all tasks, variable importance rankings for feature selection were carried out for each data type separately (CorEx plasma, CorEx brain, plasma, brain). Within each subgroup analysis, we combined the top 10 measures for each data type and performed the same feature importance scheme to determine the relative importance of these selected features across the joint set of all top measures. All statistical analyses were performed using R, version 3.4.4 (R Core Team, 2013).

### Disease Prediction

Using the top features across all data types from our feature selection step, we performed disease conversion prediction using gradient boosting machine (GBM) learning (Freund et al., 1997; Friedman, 2001). This technique is based on the principle that combined learners (decision trees) can outperform single learners, and thus is aligned with the combinatoric network approach of CorEx. We used GBM for predicting diagnosis as it can be more robust to collinearity issues than other common machine learning models, and therefore suited for prediction with both CorEx factors and the corresponding original features. In GBM, trees are grown sequentially to reduce the errors of the previous trees, but the residuals are resampled, and a fraction of the data is available at each iteration to reduce overfitting. Learning is regularized through shrinkage on the learning rate. For our prediction tasks, data was randomly stratified by diagnosis with 70% used for training and 30% used for hold-out testing. Parameters were optimized using a grid-search under a 10-times repeated 10-fold cross-validation framework on the training data only. Results on hold-out test data were used to determine the average accuracies across the repeated cross-validations. Given the disproportionate number of individuals between diagnostic groups, we applied model weights to balance the groups for disease predictions. Area under the receiver operating curve (AUC) was calculated to determine the models’ overall discriminative power on the test data. Within each diagnostic group prediction, we iteratively applied these methods to identify which combinations of feature types yielded the best predictive AUC, and to understand how CorEx features contributed to the overall results.

To estimate the stability and relative contributions of the individual variables and CorEx latent factors in predicting continuous outcome measures, we performed feature selection and ran 1,000 bootstrap stepwise regressions for temporal lobe TBM, ventricle TBM, and the longitudinal cognitive composite score. For cognitive predictions we included all data types as input features (CorEx plasma, CorEx brain, plasma, brain). To identify peripheral markers specific to brain atrophy, input features for TBM predictions were limited to CorEx plasma and original plasma measures.

## Results

Demographics for the 829 ADNI phase-1 participants in the current study are categorized by baseline diagnosis and sex in Table 1.

**Table 1:** Demographics.

	Cognitively normal		Mild cognitive impairment		Alzheimer’s disease	
			
	Females	Males	Females	Males	Females	Males
*N*	112	119	140	258	98	102
Age (*SD*)	76.09 (4.72)	75.71 (5.27)	73.68 (7.49)	75.38 (7.29)	75.02 (7.95)	76.05 (7.47)
Education (*SD*)	15.28 (2.8)	16.79 (2.69)	15.22 (2.99)	15.93 (3.0)	13.82 (2.96)	15.41 (3.33)
APOE4 Count (0/1/2)	83/27/2	87/29/3	62/58/20	123/108/27	37/44/17	30/52/20
MMSE (*SD*)	29.22 (0.94)	29.01 (1.05)	26.85 (1.78)	27.10 (1.77)	23.24 (2.01)	23.32 (2.09)
Logical Memory (*SD*)	14.28 (3.33)	13.33 (3.58)	7.05 (3.28)	7.17 (3.09)	4.13 (2.85)	3.81 (2.83)
Forgetting Rate (*SD*)	32.48 (7.77)	28.40 (8.25)	23.09 (8.74)	19.92 (7.32)	16.98 (7.64)	14.95 (6.83)
Delayed Recall	13.40 (2.06)	12.41 (2.81)	9.71 (4.01)	9.66 (3.42)	7.08 (3.91)	7.26 (4.05)
Learning Over Trials	1.81 (0.49)	2.12 (2.89)	1.51 (0.46)	1.58 (0.53)	1.40 (0.59)	1.46 (0.59)
Trails B	44.72 (1.95)	44.27 (1.56)	77.18 (1.91)	71.06 (1.79)	86.37 (1.85)	88.39 (1.89)
CDR-SOB	0.05 (0.15)	0.0 (0.05)	1.61 (0.80)	1.59 (0.93)	4.57 (1.65)	4.19 (1.58)
ADAS-13	8.63 (4.29)	10.28 (3.99)	18.91 (6.83)	18.45 (5.93)	28.85 (7.52)	29.22 (7.64)


*Basic demographic information for the ADNI participants included here, grouped by baseline diagnosis and sex.*

### CorEx Networks

**FIGURE 2:**
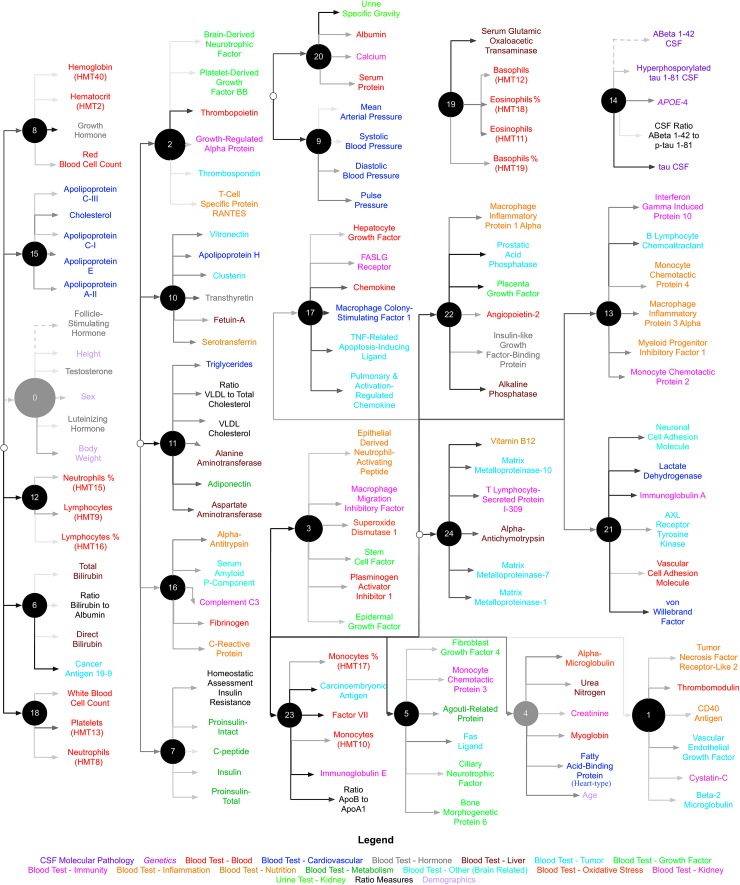
Hierarchical representation of biomarkers constructed by CorEx. Using CorEx across a panel of >200 markers of plasma, demographic, and CSF measures, we constructed a hierarchical network based on the joint information shared between measures. We identified 25 latent factors that represent the optimal total correlation across measures and factors. Latent factors are represented by circular nodes and numbered accordingly. Colors indicate variable type, as defined in the bottom legend, and gray shaded edges reflect the amount of mutual information shared between connecting nodes, where darker edges indicate more shared information. The size of each node is a function of the amount of mutual information shared among the connected variables.

**FIGURE 3:**
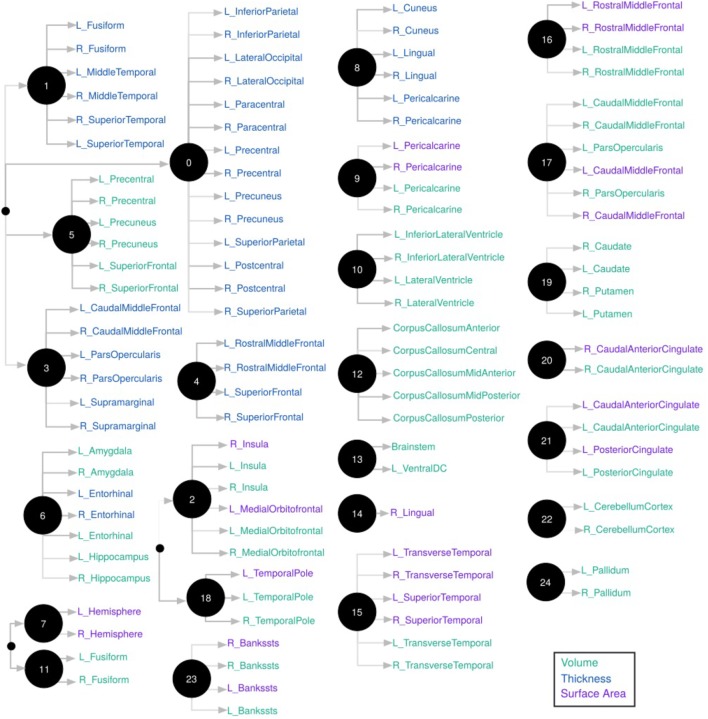
Hierarchical representation of brain measures constructed by CorEx. Using CorEx across a panel of >200 residualized gray matter measures we constructed a hierarchical network based on the joint information shared between measures. We identified a set of 25 latent factors that represent optimal total correlation across measures and factors. Latent factors are represented by circular nodes and numbered accordingly. The colors indicate measurement type, defined in the legend on the bottom right, with thickness measures in *blue*, cortical volumes in *green*, and surface area in *purple*. Gray shaded edges reflect the amount of mutual information shared between connecting nodes; darker edges indicate more shared information. Only the top measurements are shown for each node.

We used CorEx to learn low-dimensional representations and reconstruct meaningful biological and hierarchical structures using data across plasma, CSF, genetic, and demographic measures, and separately for cortical brain measures. We then built more robust predictors that we display in the form of a tree-based network in Figures 2, 3. Measures are labeled with text and color-coded based on the measurement type, indicated in the key. Latent factors are illustrated as ‘nodes’ in the graph (and factors at the first level of the hierarchy (*k* = 1 as in Supplementary Figure 1) are numbered 0, …, 24). Links reflect learned functional relationships between variables and the gray shade of an edge reflects the shared mutual information (darker indicates more shared mutual information). The size of a latent factor node is based on the amount of multivariate mutual information among its children nodes. Hierarchical groups constructed by CorEx were biologically coherent, such as a cluster of apolipoproteins (factor 15) and the hallmarks of AD (factor 14).

### Predicting Cognitive Decline

**FIGURE 4:**
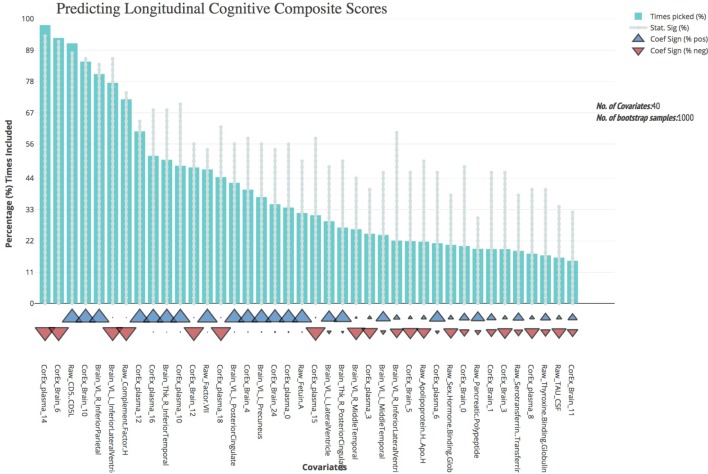
Bootstrap regression results for a longitudinal cognitive composite score. As outlined in the Methods, we created a longitudinal cognitive composite score broadly encompassing executive function, orientation, attention, verbal, short-term, and working memory. Scores are based on change from baseline to 1-year follow-up and were significantly associated with progression to AD within the baseline CN and MCI groups (*p* < 0.001) and of AD longitudinally across all groups (*p* < 0.001). To understand the most predictive features of this score, we performed feature selection across feature types and then ran bootstrap stepwise regressions using 1,000 permutations. The top features are shown on the left; arrows underneath the histograms indicate the direction of effect for each measure. A prefix of “Raw” and “Brain” are used to denote the original measures, while “CorEx” indicates the transformed factors identified using CorEx. Corresponding regions included in the CorEx factors are outlined in Figures 2, 3.

We first validated our cognitive composite score using logistic regression, adjusting for age, sex, education, and APOE4 status and found the composite score significantly predicted progression to AD with both broad and specific group classification (non-AD vs. AD, *p* < 0.001; baseline CN and MCI groups, *p* < 0.001). Bootstrapped stepwise regressions showed that the CorEx latent factor representing AD hallmarks (factor 14) was the strongest predictor of our longitudinal cognitive composite, with a negative direction of effect. Other important features included CD5 Molecule Like (CD5L), and the CorEx latent brain factor (factor 6) that includes thickness and volume measures of the limbic lobe (amygdala, hippocampus, and entorhinal cortex). Several CorEx latent plasma factors (e.g., factor 10, 12, 14, 16) showed greater predictability than the individual measures, as indicated by the number of times the features were selected across bootstrap samples (Figure 4).

### Predicting Brain Atrophy

**FIGURE 5:**
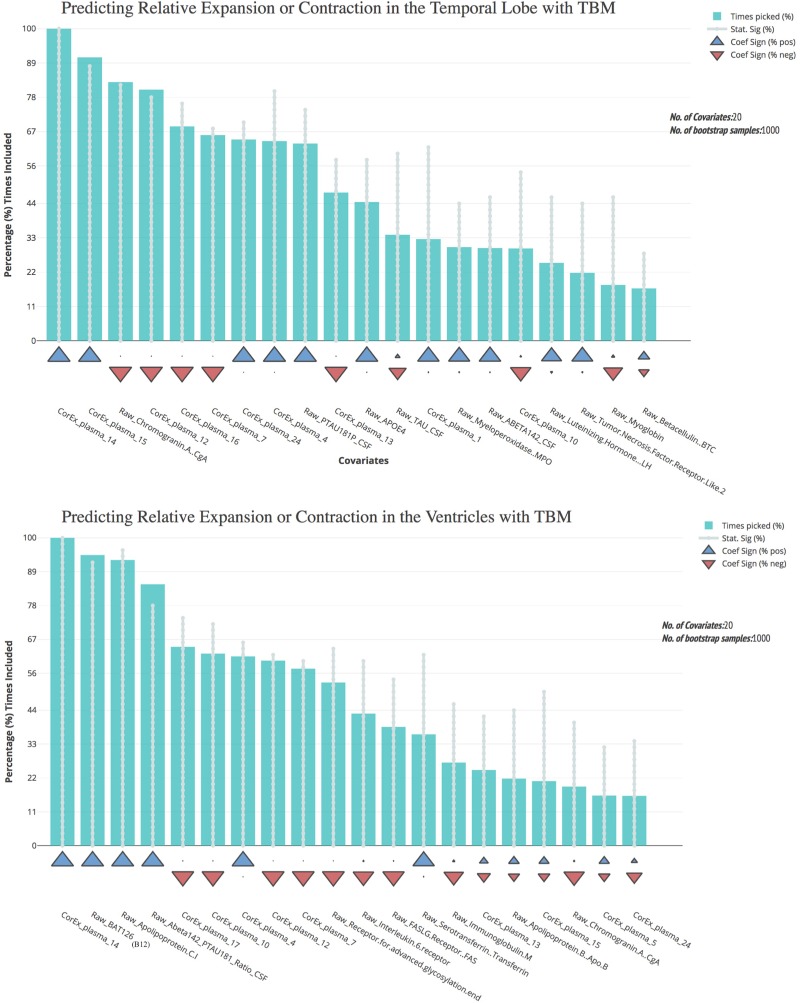
Bootstrap regression results for atrophy measures using tensor-based morphometry in the temporal lobes **(top)** and ventricles **(bottom)**. As outlined in the Methods, we computed tensor-based morphometry (TBM) measures of atrophy between baseline scanning and 1-year follow-up. To understand the most predictive features of these atrophy measures, we performed feature selection using CorEx plasma and the original plasma measures, and then ran bootstrap stepwise regressions using 1,000 permutations for each TBM measure. The top features are shown on the left; arrows indicate the direction of effect for each measure. A prefix of “Raw” and “Brain” are used to denote the original measures, while “CorEx” indicates the transformed factors identified using CorEx. Corresponding regions included in the CorEx factors are outlined in Figures 2, 3.

Most of the CorEx factors that were maintained following feature selection were the same for the TBM measures of brain change over time (Figure 5). Latent factors 4, 7, 12, and 14 were the strongest predictors for both temporal lobe and ventricle TBM measures. However, CorEx latent factors 5 and 17 were maintained only for the ventricles, and CorEx latent factors 1, 16, and 24, were maintained only for the temporal lobe. All measures included in these factors included proteins involved in the immune response, such as cell surface markers CD40, complement C3, monocyte chemotactic protein 3, and macrophage colony stimulating factor. There was overlap among some but not all of the top CorEx latent factors and the original features they represent. The CorEx latent factor including the classic AD hallmarks (factor 14) was the single most predictive measure for both TBM measures. Latent factor 15 – a cluster of apolipoproteins and cholesterol measures - was also highly predictive for temporal lobe TBM, even though the individual measures were not. Chromogranin A was a strong individual predictor of temporal lobe TBM, but a weaker predictor for the ventricles. Finally, of the features selected with the bootstrap stepwise regression at least 50% of the time, CorEx plasma factors comprised 60 and 80% of the predictors for ventricle and temporal lobe TBM, respectively; corresponding to greater predictive performance overall for the CorEx factors than for the original measures.

### Predicting Longitudinal Diagnosis

**FIGURE 6:**
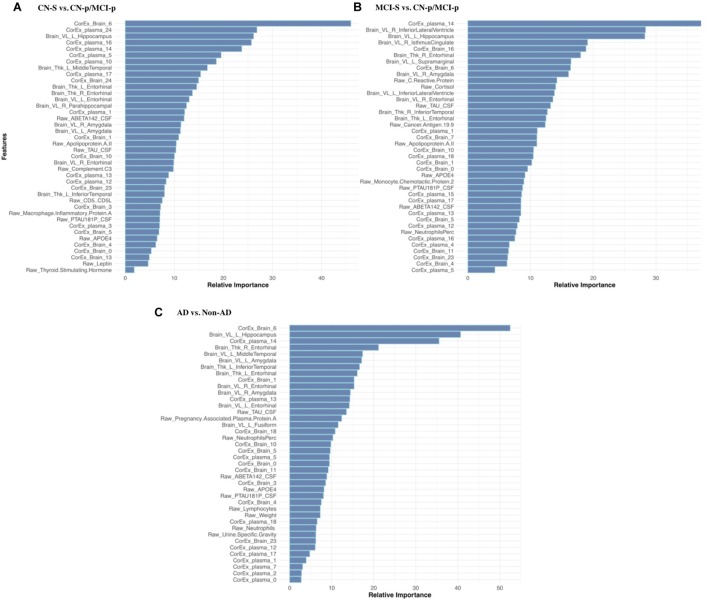
Feature selection results for longitudinal prediction of AD. Using our ensemble feature selection technique outlined in the Methods section, we identified the top 10 features within each feature type (CorEx plasma, CorEx brain, plasma, brain) across each of our three diagnostic groups used for prediction: **(A)** CN-s vs. CN-p/MCI-p, **(B)** MCI-s vs. CN-p/MCI-p, **(C)** non-AD vs. AD. We then combined the top 10 features across feature types and within diagnostic groups to identify the relative importance of these 40 features for each diagnostic group. A prefix of “Raw” and “Brain” are used to denote the original measures, while “CorEx” indicates the transformed factors identified using CorEx. Latent factors are defined in Figure 2 for CorEx plasma measures, and Figure 3 for CorEx brain measures.

**FIGURE 7:**
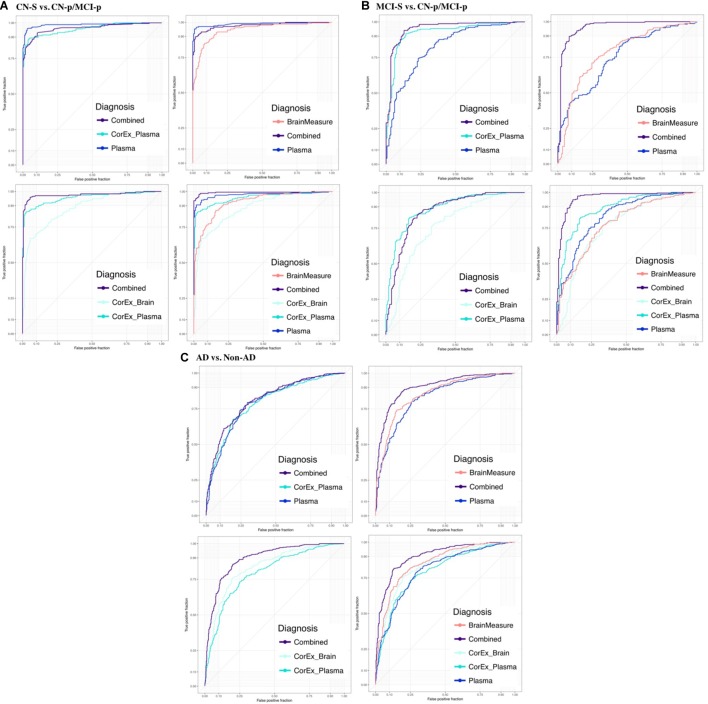
Receiver operating characteristic plots for the average testing sets across diagnostic groups and feature types. We show the area under the ROC curve (AUC) averaged across testing repeats for **(A)** CN-s vs. CN-p/MCI-p, **(B)** MCI-s vs. CN-p/MCI-p, **(C)** non-AD vs. AD. We denote the progressing groups by “CN-p” or “MCI-p”. We included individual groups of measures (plasma, hallmarks of AD, demographics, and gray matter measures) and CorEx features in the prediction and explored different combinations of these feature groups to understand the benefit of including CorEx factors. Across diagnostic groups we largely see improved AUC with inclusion of CorEx factors. CorEx factors are outlined in Figures 2, 3.

Feature selection results showing the relative importance of the top CorEx factors and the individual features for predicting longitudinal progression are presented in Figure 6. Using our ensemble feature selection technique, the top features performed well across diagnostic subgroup analyses, with an accuracy ranging from 71.9–88.2% (Table 2). Receiver operating characteristic (ROC) curves (Figure 7) show how the testing performance was affected by different combinations of feature types.

**Table 2:** Tests of prediction results using gradient boosting machines across diagnostic groups and feature types.

		Stable CN vs. Progressors				Stable MCI vs. Progressors				Longitudinal AD vs. No AD			
				
		AUC %	ACC %	SEN %	SPEC %	AUC %	ACC %	SEN %	SPEC %	AUC %	ACC %	SEN %	SPEC %
Plasma + CorEx_Plasma	Plasma	99.00	90.76	85.71	94.29	82.00	66.67	34.09	87.14	80.00	68.44	61.74	74.42
	CorEx_Plasma	96.00	89.92	91.84	88.57	92.00	63.16	40.91	77.14	79.00	64.34	58.26	69.77
	Combined	96.00	89.99	93.88	87.14	95.00	67.54	47.73	80.00	81.00	71.72	66.96	75.97
CorEx_Brain + CorEx_Plasma	CorEx_Brain	88.00	76.47	59.18	88.57	76.00	68.42	31.82	91.43	83.00	76.64	72.17	80.62
	CorEx_Plasma	96.00	82.35	85.71	80.00	89.00	71.05	43.18	88.57	80.00	67.62	62.61	72.09
	Combined	98.00	87.39	85.71	88.57	87.00	73.68	50.00	88.57	89.00	82.38	77.39	86.82
Brain + Plasma	Brain	94.00	79.83	75.51	82.86	78.00	60.53	27.27	81.43	85.00	74.59	76.52	72.87
	Plasma	99.00	97.48	97.96	97.14	75.00	67.54	20.46	97.14	83.00	68.44	65.22	71.32
	Combined	98.00	84.87	77.55	90.00	96.00	68.42	54.55	77.14	90.00	76.64	70.43	82.17
Brain + Plasma + CorEx_Brain + CorEx_Plasma	Brain	93.00	85.71	77.55	91.43	78.00	67.54	29.55	91.43	86.00	77.05	74.78	79.07
	Plasma	98.00	89.08	91.84	87.14	83.00	64.04	27.27	87.14	80.00	72.13	68.70	75.19
	CorEx_Brain	96.00	76.47	59.18	88.57	76.00	68.42	31.82	91.43	83.00	76.64	72.17	80.62
	CorEx_Plasma	96.00	82.35	85.71	80.00	89.00	71.05	43.18	88.57	80.00	67.62	62.61	72.09
	Combined	99.00	88.24	93.88	84.29	96.00	71.93	54.55	82.86	90.00	81.15	79.13	82.95


*Gradient boosted machines (GBMs) were used to predict longitudinal diagnosis for stable CN vs. CN and MCI participants who progressed to AD, stable MCI vs. CN and MCI participants who progress to AD, and all individuals who had or progressed to AD vs. those who did not (non-AD vs. AD). Results were obtained following feature selection, as shown in Figure 6, and a 70/30 split for training and testing, respectively. Additional details are outlined in the Methods. We report the area under the ROC curve (AUC) for the testing sets averaged across repeats. We included individual groups of measures (plasma, hallmarks of AD, demographics, and grey matter measures) and CorEx features in the prediction and explored different combinations of these feature groups to understand the benefit of including CorEx factors. Among the original features, plasma measures showed high AUC for the stable CN vs. CN and MCI participants who progress to AD, while brain measures were more optimal for predicting a longitudinal diagnosis of AD vs. those who do not have or develop AD.*

Results were generally improved by combining all data types, particularly for AUC metrics. We were able to predict stable cognitively normal (CN) individuals from CN and MCI individuals who progress to AD (CN-s vs. CN-p/MCI-p) with up to 88.4% test accuracy and 99% AUC. McNemar’s test identified significant differences in performance using individual plasma measures compared to latent factors of CorEx plasma measures (*p* < 4.3 × 10^-14^), while the combined feature-set was significantly different compared to using only plasma measures (*p* < 1.5 × 10^-10^), indicating optimal performance for the combined features overall. Here, CorEx plasma measures were the top individual feature type for discriminating AD from CN-p/MCI-p, with significant differences in performance compared to the original plasma measures (*p* < 0.0001) and test accuracy of 71.9% (96% AUC). Significant differences in model performance using the combined feature types was observed compared to CorEx plasma features alone (*p* < 6.3 × 10^-6^), indicating improved prediction with the combined features overall. Finally, significant differences were observed using individual structural brain measures compared to the CorEx brain measures for discriminating non-AD and AD groups (*p* < 1.8 × 10^-9^), while the combined set of features performed the best overall compared to using structural brain measures alone (*p* < 5.6 × 10^-15^).

The CorEx plasma factor of AD hallmarks (factor 14) was consistently more predictive across all analyses than any individual classical AD hallmark. In the CN-s vs. CN-p/MCI-p analysis, the relative importance of CorEx plasma factor 14 was nearly twice that of Aβ1-42 and CSF tau CSF, and this difference was larger for the MCI-s vs. CN-p/MCI-p and the AD vs. non-AD analyses. However, this factor was not the most important factor overall in the CN-s vs. CN-p/MCI-p analysis. Interestingly, CSF total tau was more important than phosphorylated tau in each of these analyses. Not all of the important individual features were contained within the most important CorEx plasma factors, such as apolipoprotein AII for the CN-s vs. CN-p/MCI-p analyses. Likewise, some of the top individual features were not captured within CorEx factors overall, such as CD5L, cortisol, and leptin, supporting the importance of combining original analytes and CorEx factors due to their somewhat independent contributions to predicting AD.

## Discussion

Crosstalk between the brain and periphery is crucial for shaping neuronal survival and function, yet the etiological relevance of bi-directional interactions between neuroendocrine, neuroimmune, and bioenergetic systems, and amyloid and tau pathways for AD progression is unknown (Engelhart et al., 2004; Blennow, 2010; Chakrabarty et al., 2015; Zheng et al., 2015; Alam et al., 2016; Gonzalez et al., 2017). Here we used a novel method, CorEx, to further understand this crosstalk and discover biologically relevant relationships among the hallmarks of AD, plasma markers, demographics, and MRI-derived brain measures. The latent factor representing the key hallmarks of AD such as CSF Aβ-1-42 and tau measures and APOE (factor 14) was consistently identified as a top predictor across analyses and diagnostic groups. Many features that formed a CorEx latent factor are consistent with mechanisms known to be involved in the etiology and pathological expression of AD. Specifically, CorEx discovered plasma factors of apolipoproteins involved in established biological pathways (factor 15), and two latent factors of white blood cell count measures that were connected within the CorEx network (factors 12 and 18). Importantly, these three factors were among the strongest predictors of disease progression and brain atrophy indexed through TBM.

Most CorEx latent factors of the brain depicted regions with functions that are known to overlap – such as bilateral measures for a particular region (e.g., factors 22 and 24; bilateral cerebellar cortex and pallidum, respectively), or distributed bilateral measures across the limbic lobe, encompassing both thickness and volumetric measurements (e.g., factor 6; amygdala, entorhinal cortex, hippocampus). Most other factors contained only one measurement type (surface area, volume, or thickness), or a combination of surface area and volume. Thickness measures were less likely to be combined with surface area or volume measures within a factor, in line with prior studies showing that cortical thickness exhibits roughly linear decreases with age in most brain regions, whereas cortical volume and surface area show overlapping curvilinear trajectories (Sanabria-Diaz et al., 2010; Rimol et al., 2012; Storsve et al., 2014).

Although the brain measures grouped within a factor were generally as expected from systems biology, certain plasma measures were not. For instance, we would not have expected that apolipoprotein H or clusterin would be grouped in a separate factor from other apolipoproteins, given their shared role in lipid transport, or that the ratio of apolipoprotein B to apolipoprotein A1 would be separate either, and that this ratio would be within a factor consisting primarily of immune markers such as monocytes and immunoglobulin E. However, in line with this, prior studies have shown that regulatory T cells promote the clearance of apolipoprotein B-containing lipoproteins via increasing the expression of sortilin-1 and lipid-modifying enzymes by the liver, pointing to the interplay between the immune system, lipid metabolism, and the liver, which may synergistically mediate risk for AD (Getz and Reardon, 2014). Here we found apolipoprotein B to be predictive of expansion of the ventricles, while Factor VII in the same CorEx factor was predictive of the longitudinal cognitive composite measure.

As shown in Figure 6, the most important features in predicting progression to AD varied depending on the comparison group (i.e., stable cognitively normal, stable MCI, or more generally individuals who do not progress to AD). Volume of the left hippocampus – and the latent factor containing medial temporal and amygdala structures (factor 6) were highly important for predicting clinical progression from cognitively normal status (CN-s vs. CN-p/MCI-p), and those with MCI (MCI-s vs. CN-p/MCI-p). Conversely, latent factors of plasma markers were generally the strongest predictors of progression to AD in CN/MCI participants (CN-s vs. CN-p/MCI-p), suggesting that they may be sensitive markers of early disease progression. This supports the utility of plasma measures as an early biomarker tool, and corroborates the crosstalk between the peripheral and central nervous system in mediating risk for disease. Although previously associated with AD, certain plasma markers were not retained as latent factors following feature selection for any of our analyses (e.g., latent plasma factor 6 and 9), which includes measures of bilirubin and blood pressure related measures respectively, and factor 11 which includes very-low-density lipoproteins, triglycerides, and adiponectin. These findings may suggest that these particular markers are only nominally associated with AD, and that compared to other measures included here, they may not provide additional predictive power.

The most important CorEx factor predicting our cognitive composite were volume and thickness measures of the medial temporal lobe (factor 6), which are among the earliest regions to exhibit neuronal degeneration, neurofibrillary tangle deposition, and accumulation of amyloid in AD (Braak and Braak, 1991; Scheff and Price, 2003). The most important plasma measures for our cognitive composite were the latent factor representing the hallmarks of AD, and CD5L, a soluble immune effector expressed primarily by mature macrophages that is involved in fatty acid metabolism and lipid biosynthesis (Sanjurjo et al., 2015). Importantly, CD5L is involved in regulating the inflammatory response to pathogens and in developing and maintening the lymphoid compartment and may have additional relevance to AD through its regulatory roles of apoptosis and autophagy (Miyazaki et al., 1999; Nixon and Yang, 2011). Additionally, although identified in a small sample of 78 subjects, plasma levels of this protein have been associated with neocortical amyloid burden (Ashton et al., 2015). Given the established association between Th17 cells and CD5L, and the interaction between Th17 cells and neurodegeneration, these results suggest follow-up studies elucidating the specific role for CD5L in AD is warranted (Zhang et al., 2013; Wang et al., 2015).

CorEx discovered latent factors that showed greater predictive accuracy for AD progression than the individual hallmarks of AD, but no individual plasma marker showed greater importance than the latent factor representing the established AD hallmarks. This points to the importance of considering plasma markers jointly. Other factors were consistently important for predicting clinical progression across all of our analyses, such as chemokines and white blood cell markers of the innate and adaptive immune system (factors 12 and 13). An impaired immune response to toxic amyloidogenic substances may make it easier for amyloid to accumulate in the brain (Richartz-Salzburger et al., 2007). Likewise, amyloid precursor protein (APP) mRNA expression is increased in peripheral lymphocytes in AD, and alterations in APP expression leading to amyloid deposition may also cause changes in peripheral immune cells, creating a feedback loop that ultimately may lead to cognitive decline and neurodegeneration (Jiang et al., 2003). A subsequent decline in lymphocyte levels and lymphocyte percentages may indicate immune dysregulation or immunosenescence in AD. Similarly, neutrophils defend tissue against invading pathogens during sterile inflammation and increases in neutrophil levels are also associated with blood brain barrier disruptions and chronic inflammation in AD (Phillipson and Kubes, 2011; Kolaczkowska and Kubes, 2013; Zenaro et al., 2015). Moreover, a set of dietary, immune, and inflammatory markers (factors 16 and 24) were more important predictors of early diagnosis than the hallmarks of AD (CN-s vs. CN-p/MCI-p). These factors included variables known to be modifiable by dietary and lifestyle changes, such as vitamin B12 levels and C-reactive protein (CRP). Factor 16 also included amyloid P component (AP), an important component of all amyloid deposits including those typically found in the brains of patients with AD (Yasojima et al., 2000). These relationships are also supported by prior literature. For instance, one Finnish-community dwelling longitudinal study found that serum levels of B12 were protective against AD in cognitively normal individuals, and that there were potential interactions with homocysteine serum levels, another modifiable marker (Hooshmand et al., 2010). Likewise, CRP is predictive of later cognitive decline in mid-life (Laurin et al., 2009). Moreover, CRP and AP are both acute phase proteins of the innate immune response and colocalize in neurons in individuals with AD (Steel and Whitehead, 1994). As part of the innate immune response, CRP and AP activate the classical complement pathway in an antibody independent fashion, and indeed factor 16 includes complement C3, further supporting the network approach achieved with CorEx. Activation of complement is important for opsonizing targets for phagocytosis and the subsequent destruction of pathogens, such as amyloid beta plaques (Hicks et al., 1992; Wolbink et al., 1996). The autodestructive association of CRP and AP with activated complement fragments attached to host tissue has been seen in degenerative conditions, such as atherosclerosis and AD (Lagrand et al., 1997; Torzewski et al., 1998; McGeer et al., 2001).

A few aspects of the data on individual predictors deserve comment. Firstly, our data are based on a variable length of follow-up (roughly 1–5 years), which optimizes the available study information. A homogeneous follow-up interval would not have been ideal as it would have reduced the number of available participants progressing to AD and predictions based on a smaller sample size may generalize poorly. Secondly, the cross-sectional design of the plasma markers is an important limitation and we could not follow up on the potential cyclical or prandial changes of some of these measures, so we are unable to make conclusions about the specific trajectory of these measures. It will also be important to replicate these findings in future cohorts. We did not specifically evaluate other diseases of old age which may be associated with altered plasma markers. However, all individuals were neurologically normal aside from Alzheimer’s disease, and conditions such as cardiovascular disease and diabetes are typically defined using measures included in our analysis. Additionally, sex was used as a demographic variable included in the plasma measures when constructing the CorEx plasma latent factors. As sex mediates multiple pathways involved in AD risk, such as APOE, some of these factors may have been grouped differently if we had constructed the factors separately for women and men (Riedel et al., 2016; Fisher et al., 2018). Indeed, the CorEx factor 0 – which includes sex – was among the most important features for predicting longitudinal diagnosis of AD (non-AD vs. AD), though body weight and hormone levels also contained in this factor may be driving this association. Finally, although there is a disparate number of women and men with MCI within ADNI, we did not find CorEx factor 0 to be predictive of progression within this group. Future work will seek to address potential confounds and determine sex-specific risk profiles.

## Summary

We tested CorEx, a novel model-free data-driven approach to combine relevant groups of >400 potential biomarkers from brain imaging, genetics, plasma, and demographic information. We discovered a small set of tractable relationships in 829 participants across the trajectory from normal cognition to MCI and AD. While the relationships between some of the measures have been previously documented and several measures were known to be associated with AD, the clustering achieved with CorEx provides more direct evidence for a network of related measures and how these measures jointly predict disease progression, brain atrophy, and cognitive decline. These results also demonstrate the power of CorEx to identify clusters of variables that involve synergistic and coherent sets of the original features, revealing stronger combinations of variables that may be only weakly predictive when examined as individual predictors. Our results point to the consistent importance of amyloid and tau across the disease trajectory, but also to the timepoint specific contributions of the immune and inflammatory systems, and to the role of cardiovascular health, hormone levels and lipid and glucose metabolism.

## Author Contributions

BR conceived and designed the analysis, analyzed and interpreted the results, and drafted the manuscript. MD, AM, LS, and PT assisted with analysis and interpretation of data. GVS and AG worked on algorithm development for CorEx. All authors assisted with editing of the manuscript.

## Conflict of Interest Statement

The authors declare that the research was conducted in the absence of any commercial or financial relationships that could be construed as a potential conflict of interest.

## Footnotes

1 http://www.adni-info.org/

2 https://adni.loni.usc.edu/wp-content/uploads/2010/11/BC_Plasma_Proteomics_ Data_Primer.pdf

3 https://surfer.nmr.mgh.harvard.edu/

4 https://github.com/gregversteeg/Bio_CorEx
