# Supplementary material for: Uncovering Biologically Coherent Peripheral Signatures of Health and Risk for Alzheimer’s Disease in the Aging Brain
Source: Front Aging Neurosci. 2018 Nov 29;10:390. doi: 10.3389/fnagi.2018.00390 (PMC6283260; doi:10.3389/fnagi.2018.00390)
Supplement: Supplementary file 1 [file Image_1.pdf]

Supplementary Material

Uncovering Biologically Coherent Peripheral Signatures of Health and Risk for Alzheimer's Disease in the Aging Brain

Brandalyn C. Riedel[1], Madelaine Daianu[1], Greg Ver Steeg[2], Adam Mezher[1,†], Lauren E. Salminen[1], Aram Galstyan[2], Paul M. Thompson[1,3], and the Alzheimer's Disease Neuroimaging Initiative*

*** Correspondence:** Paul M. Thompson pthomp@usc.edu

1 Supplementary Data

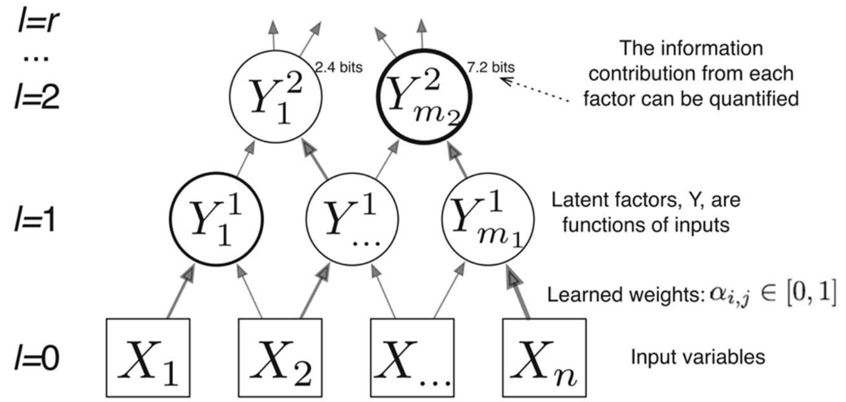

Supplementary Figure 1. Example CorEx mathematical representation of latent factor construction. The graphical model depicted is optimized to learn latent factors, Y, that minimize TC(X|Y) + TC(Y) = 0 (Ver Steeg, 2017), where TC = total correlation, X are the input variables $X_1 \ldots X_N$, and Y are the constructed latent factors. In other words, starting from the top layer, each layer learns to explain dependence in the layer below. The lowest layer represented $X_1 \ldots X_N$ signify the original observed measures, while all subsequent layers represent latent factors learned through CorEx.
